# Methods integrating innate and adaptive immune responses in human *in vitro* immunization assays

**DOI:** 10.3389/fimmu.2025.1584852

**Published:** 2025-05-21

**Authors:** Tetiana Y. Bowley, Kiersten D. Lenz, Apoorv Shanker, Jessica Z. Kubicek-Sutherland

**Affiliations:** Physical Chemistry and Applied Spectroscopy, Chemistry Division, Los Alamos National Laboratory, Los Alamos, NM, United States

**Keywords:** *in vitro* immunization, innate immunity, adaptive immunity, whole blood assay, microphysiological human tissue construct assay, monocyte-derived dendritic cell assay, dendritic cell-t cell interface assay, vaccine development

## Abstract

Rapid vaccine development and innovative immunotherapeutics are critical in the fight against emerging outbreaks and global pandemic threats, yet the high costs and prolonged timelines for developing new vaccines underscore the urgent need for robust, predictive pre-clinical testing platforms. The rapid down-selection of vaccine candidates and identification of optimal vaccine formulations can be performed using human *in vitro* immunization (IVI) assays that recapitulate the complex interactions of the innate and adaptive human immune response. In this review, we present a comprehensive evaluation of three key IVI platforms: the whole blood assay (WBA), monocyte-derived dendritic cell (MoDC) assay with dendritic cell-T cell interface assay (DTI), and the microphysiological human tissue construct assay (HTC). The WBA offers a cost-effective and straightforward approach, while the MoDC + DTI system represents the current gold standard for balancing experimental efficiency with immunological complexity. The HTC assay, by mimicking both spatial and temporal aspects of immune interactions, provides enhanced physiological relevance. We discuss the methodological advantages and limitations of each platform, explore their roles in rapid vaccine candidate screening, and propose strategies for integrating these assays with complementary *in vivo* models. These insights pave the way for refining IVI assays and accelerating the translational pipeline for next-generation vaccines and immunotherapies.

## 1 Introduction

The rapid development of novel vaccines requires *in vitro* testing techniques capable of reproducing the complexity of human immunology. A vaccine is recognized by the immune system as a foreign antigen similar to natural pathogen infection (1). Innate immunity is the first line of defense that in turn trains adaptive immunity by modulating the quantity and activation status of long-term T and B cells (2). It is currently estimated to take between 10–15 years for a new vaccine to be developed, with much of that time spent on pre-clinical trials including animal studies (3). During vaccine development, several challenges are faced in generating an optimal vaccine to a specific pathogen including pathogen variability, optimizing formulations to elicit a strong and lasting immune response, minimizing side effects, and overcoming human variability (4). Human *in vitro* immunization (IVI) assays offer a time-saving platform to test the efficacy of new vaccine candidates by providing a physiologically relevant environment to screen potential vaccine formulations. IVI assays are a unique tool that allows researchers to isolate specific immune cell populations and reconstitute immune responses to a pathogen or a vaccine in a laboratory setting. Human blood or peripheral blood mononuclear cells (PBMCs) are used for IVI assays to measure human responses. Murine IVI assays can be more directly comparable to preclinical *in vivo* studies performed in mice but do not always recapitulate a human response (5). *In vivo* models have the benefit of capturing the complexity of biological systems allowing evaluation of the immune response in a natural environment (6). Although *in vivo* models can provide physiological relevance, they can have high costs, limited availability for the type of animal required, longer experimental timelines, and ethical considerations (7). Initial vaccine candidate screens using IVI assays can provide a more accurate assessment of human immune responses, reduce costs and timeline associated with screening candidate vaccines, and avoid unnecessary animal experimentation.

**Figure 1:**
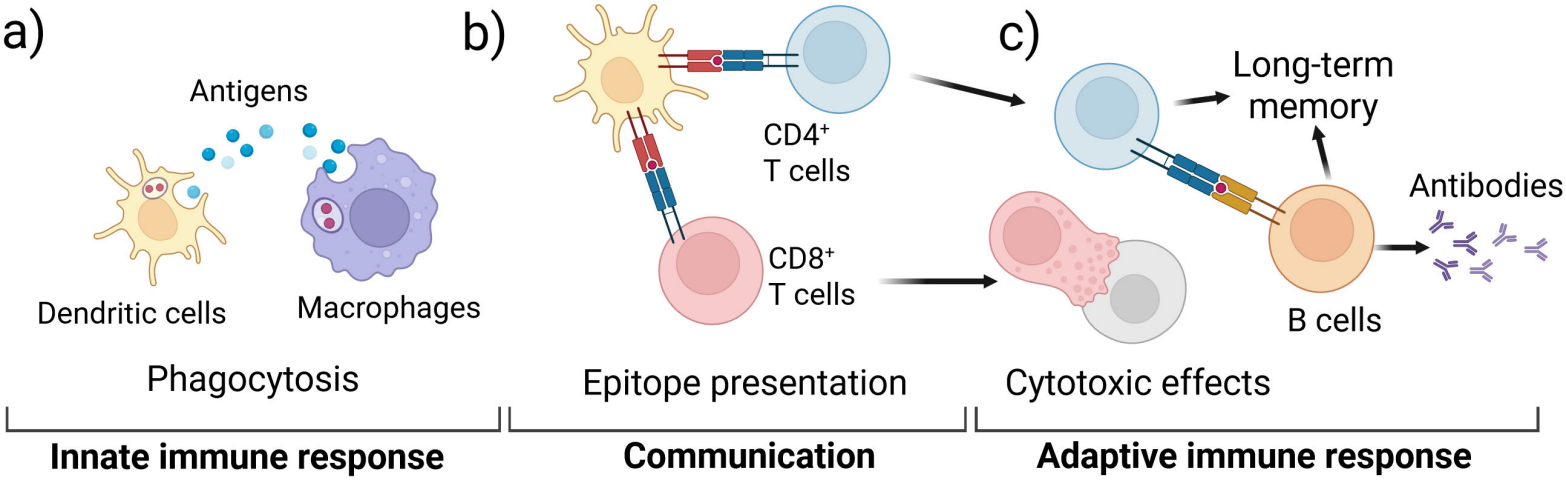
Schematic of key immune responses recapitulated in IVI assays; **(a)** the innate immune system initially responds to a vaccine antigen via phagocytosis in DCs and macrophages; **(b)** the phagocytotic cells digest and present antigen epitopes to T cells of the adaptive immune system; **(c)** activated T cells activate B cells that produce specific antibodies and contribute to long-term memory.

For an IVI assay to successfully recapitulate the complex human immune system and be useful to the vaccine development pipeline, both the innate and adaptive immune responses must be captured as these responses work together to provide protective immunity (**Figure 1**). Innate immune cells include neutrophils, macrophages, monocytes, dendritic cells (DCs), natural killer (NK) cells, mast cells, eosinophils, and basophils (8–10). Upon exposure to a vaccine antigen, the innate immune system is activated to elicit an immediate but non-specific anti-microbial immune response (9). Macrophages and DCs (**Figure 1a**) ingest and process antigens, using major histocompatibility complex (MHC) proteins to present epitopes to T cells of the adaptive immune system (**Figure 1b**) (11). Adaptive immune cells include T cells and B cells that create a specific response and memory to a pathogen (**Figure 1c**) (12, 13). The phagocytic cells of the innate immune system present epitopes to both CD4^+^ and CD8^+^ T cells, activating them to perform their functions (10, 13–15). CD8+ T cells, also called cytotoxic T cells, are responsible for killing pathogen-infected cells. CD4^+^ T cells, also called helper T cells, go on to stimulate B cells primarily in the secondary lymphoid organs. The activated B cells produce antibodies to aid in long term immunity along with memory T cells (**Figure 1c**) (13).

The source of immune cells for IVI assays is most often whole blood or PBMCs (16–18). Whole blood contains a variety of cells including erythrocytes, leukocytes, and thrombocytes (platelets), as well as blood plasma (water, ions, hormones, etc.). An advantage of using whole blood is that all the major immune cells are present; however, the immune cells are present at lower concentrations and the sample must be processed to isolate cells of interest (18, 19). PBMCs consist only of monocytes, DCs, and lymphocytes; and they are isolated from whole blood samples via centrifugation where they end up in the buffy coat (18, 19). PBMCs can be cryopreserved and analyzed at a later point in time, while whole blood must be processed immediately to retain functionality of granulocytes (18). PBMC cryopreservation is widely used in human immunological studies (20–22). It is known that PBMC cryopreservation influences cellular viability, phenotype, and functional state of cells due to intracellular formation of ice crystals during the freezing process (23). The benefits are that whole blood and PMBCs are both easily accessible from clinics and allow for longitudinal studies from the same donor.

DCs are a critical component of the immune response to vaccines (14, 24–27). There are two stages of DC maturation: mature and immature (28, 29). Immature DCs are scattered throughout the body, where they constantly sample the surrounding environment for pathogens and vaccine antigens by means of endocytosis (30, 31). Once they encounter and capture a pathogen or antigen, DCs relocate to secondary lymphoid organs where they mature to process and present the antigen on their surface (31–33). DCs are the most effective antigen-presenting cells (APCs) in activating naïve T cells (31, 34–36). Treated DCs must be present in order to train T and B cells. Treatment of isolated T and B cells with an antigen or a vaccine will not produce an appropriate immune response in the absence of DCs (37). The T cell response occurs only after three signals are received: T cell receptor binding to the MHC-peptide complex, CD28 on T cell binding with either CD80 or CD86 on the DCs and cytokines produced by the DCs (38). A single DC can stimulate several antigen-specific T cells (39, 40).

The use of DCs is ubiquitous in immunological studies, including IVI assays, but there are challenges associated with their use. One challenge is that DCs comprise only 1-2% of PBMCs, making it difficult to obtain enough cells for experiments (41, 42). Another challenge is that immortalized human DC cell lines (e.g., MUTZ-3) have functional and transcriptional defects and produce impaired immune responses to stimulators such as lipopolysaccharide (LPS) (43, 44). The results of DC-based *in vitro* experiments cannot be relied on to guide vaccine development unless the response measured is similar to a natural immune response.

Monocyte-derived dendritic cells (MoDCs) are a distinct subset of DCs that play a critical role in inflammation and infection (45, 46). Mature MoDCs release cytokines and chemokines to attract immune cells to the infection site (31). MoDCs also activate cytotoxic CD8^+^ T cells through antigen presentation (47, 48). MoDCs are used extensively in immunology studies. They are considered the gold standard for DC-based *in vitro* experiments; however, MoDCs generated *in vitro* may differ significantly from natural MoDCs (49, 50).

*In vitro* assays can be useful tools to measure human immune responses to different pathogens and vaccine antigens aiding in the development of vaccines and immunotherapeutics (51, 52). Several physiologically relevant IVI assays have been developed to recapitulate both the innate and adaptive immune responses. However, the complexity of the human immune system is difficult to reproduce, and therefore each IVI assay has its challenges. The goal of this review is to describe the components of various IVIs, compare the advantages and limitations of each, and to identify the potential use of these assays in vaccine development. The focus of this review is on WBA, MoDC-DTI, and HTC IVI assays since they directly build upon each other to add biological complexity. The direct comparison of these IVI assay supports the selection of the most appropriate assay depending on research requirements (**Table 1**). More complex IVI assays (MoDC-DTI and HTC assays) provide a stronger immune response, but other factors may impact a researcher’s choice including costs, reagent, and time constraints (**Table 1**).

**Table 1:** Advantages and limitations of the IVI assays.

Platform	Advantages	Limitations
WBA	• High number of viable immune cells • Presence of all immune cell populations • Blood requires minimal sample processing • Low cost • Low to moderate expertise required	• Short shelf-life of the samples • Samples must be used immediately, no cryopreservation • Low number of dendritic cells (<1%) • No spatial or temporal features of immune system interactions
MoDC + DTI	• Cryopreservation of cells allows for sample preservation and flexibility in testing schedule • Conservation of phenotypic and functional characteristics of DCs • Incorporates temporal features of interactions between innate and adaptive immune system • Presence of specific interactions between autologous DC-T cell pairs	• Low throughput • Expensive compared to WBA • Time consuming experiments (up to 10 days) • Moderate expertise required • No spatial features of immune system interactions • Forced proximity and interactions of the cells
HTC	• Cryopreservation of cells allows for sample preservation and flexibility in testing schedule • DC differentiation without exogenous cytokines • Physiological relevance with both spatial and temporal features of interactions between innate and adaptive immune system	• Low throughput • Expensive compared to MoDC + DTI • Time consuming experiments (10–11 days) • High expertise required

## 2 Types of *in vitro* immunization assays

**Figure 2:**
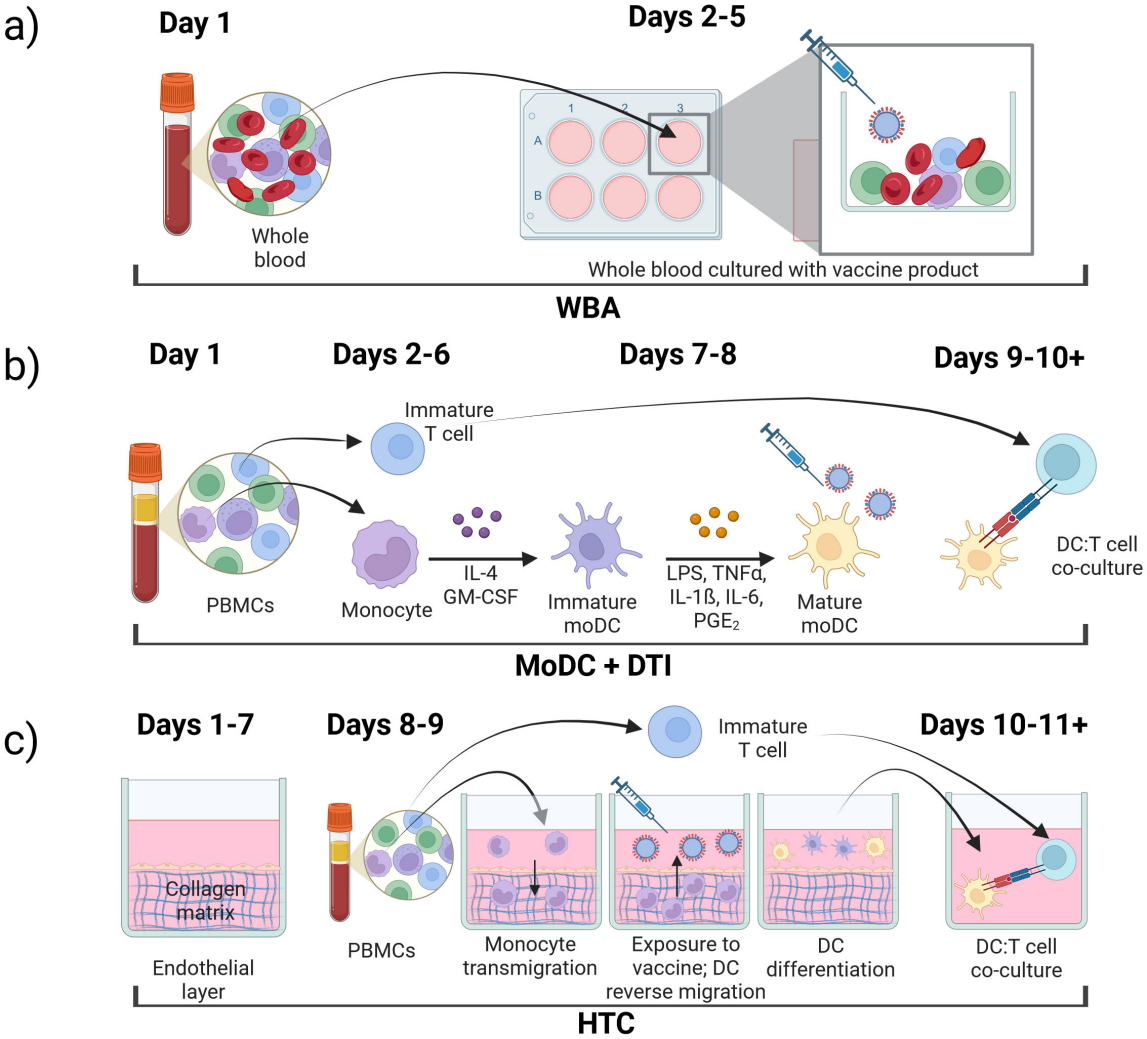
Types of *in vitro* immunization assays, **(a)** whole blood assay (WBA); **(b)** monocyte-derived dendritic cell (MoDC) assay combined with dendritic cell-T cell interface assay (DTI); **(c)** human tissue construct (HTC) assay.

The underlying approach to IVI assays is similar with varying complexity at each step: 1) immune cell isolation, 2) differentiation and antigen stimulation; 3) immune response readout(s). In the following sections, we review three prominent IVI approaches, including their experimental methods, advantages, and disadvantages (**Figure 2**, **Table 1**).

### 2.1 The whole blood assay

The whole blood assay (WBA) is an IVI assay that uses minimally processed human blood retaining all cell types of the immune system (53–56). In these assays, blood is collected in a tube containing an anticoagulant such as EDTA (18). The blood is then diluted into cell culture medium already containing the antigen or vaccine formulation to be tested and the solution is plated on a cell culture plate (18, 57, 58). After an incubation period, the cell supernatant can be collected and analyzed for secreted molecules including cytokines of interest (59). The cultures can also be processed to isolate specific cells of interest to analyze gene expression or protein expression both on the cell surface or intracellular (**Figure 2a**) (18).

The WBA has been shown to retain high viability of immune cells (55, 60). The presence of plasma makes it possible to study plasma cytokines in response to treatment (56). Plasma also eliminates the need to add serum to the growth medium, simplifying the culturing process, and creating a more natural environment. Additionally, the interactions between all cell types are preserved, making the WBA close to an *in vivo* setting. Small sample volumes (~100 µL) can be used for some assays, which is an advantage in certain laboratory settings where whole blood is limited (18). Compared to PBMC-based assays, the WBA demonstrated lower levels of leukocyte apoptosis (61), enhanced cytokine production (55, 60, 61), and lower variability between samples treated with lipopolysaccharide (LPS) (61).

One of the WBA subvariants is an assay that uses unfractionated PBMCs isolated from human blood (62). PBMCs can be cryopreserved and used retrospectively to add flexibility in the experimental plan (1). The number of cells in a blood sample can vary between samples, so PBMC counts are required to define the immune cell population in each sample (63). PBMC preparation may also introduce variability in measuring specific immune responses (63, 64). At least 10 mL of blood is required for a PBMC assay which is not always possible, especially from pediatric donors or in field setting (63). WBA assays can be performed with smaller blood volumes (<1 mL) and still provide an adequate immune response to a vaccine or a pathogen (62).

There are several limitations to using the WBA as an IVI assay. One of the main drawbacks is the short culturing period (48 hours) due to loss of nutrients and granulocyte lysis that occurs during this timeframe (55, 61). This limits studies to the early immune response versus longer-term responses. Another limitation is the need to use freshly drawn blood, since the use of cryopreserved blood introduces variation in cytokine production in response to a challenge (65, 66). This presents restrictions to researchers, requiring blood draws early in the morning followed by immediate processing, and restricting the number of donors that can be tested per day. Another challenge is the inability to mimic spatial interactions of immune cells in the human body. For instance, T cells and B cells are co-located for the entirety of the WBA. In the lymph nodes, B cells and T cells are physically separated initially, and only migrate to the edges of the lymph follicles to interact after the T cells have been activated (67). The low number of mature DCs in whole blood may also fail to stimulate a successful T cell response (68).

These observations conclude that the WBA is a good low cost starting point to evaluate immune responses quickly but does not accurately capture the spatiotemporal parameters of the immune system that may be critical factors in eliciting lasting protection to vaccination. To minimize risks of assay variability and improve reliability of results, researchers should limit physical manipulation of blood samples, avoid long term storage or cooling of blood samples, and ensure proper operating procedures and technical expertise (69, 70).

### 2.2 The monocyte-derived dendritic cell assay + DC-T cell interface assay

MoDC and DTI assays are performed consecutively to recapitulate the immune response to a specific antigen (**Figure 2b**). In the MoDC assay, monocytes are isolated from PBMCs, differentiated into MoDCs, and matured if needed. Mature MoDCs are then exposed to antigens of interest (**Figure 1a**). Next, in the DTI assay, MoDCs that were exposed to the antigen of interest are co-cultured with T-cells and the immune response is measured (**Figure 1b**). This setup is considered the gold standard for IVI experiments as it incorporates the communication between the innate and adaptive immune systems, making it more accessible when working with a limited amount of blood sample (31, 71). MoDC + DTI assays allow for enrichment of dendritic cells by a factor of 20 since the starting material is monocytes (20% of PBMC population) and not dendritic cells (1-2% of PBMC population).

#### 2.2.1 The monocyte-derived dendritic cell assay

DCs are crucial to the immune response to a foreign antigen or vaccine formulation as they are the most effective APCs (34, 35, 72). Direct isolation from PBMCs is challenging due to the low abundance of DCs (1-2%); however, monocytes are present at higher concentrations (10-20%) and can be differentiated into DCs using various growth factors in culture media (73).

First, monocytes must be isolated from PBMCs, which typically occurs by one of three methods: 1) cold aggregation; 2) centrifugation on a Percoll^®^ density gradient; or 3) magnetic bead cell enrichment (49). These methods are not interchangeable since they produce DCs with different phenotypic and functional features (74). Monocyte isolation by cold aggregation occurs when PBMCs are incubated in regular complete media (typically 90% RPMI-1640, 10% fetal bovine serum [FBS]) at 4°C with continuous agitation. Monocytes spontaneously aggregate and sediment under these conditions. Isolated monocytes can then be further enriched either by a second round of cold aggregation or centrifugation on a Percoll^®^ gradient. A Percoll^®^ density gradient is made of colloidal silica particles between 15–30 nm in diameter that are coated with polyvinylpyrrolidone (PVP) to ensure low toxicity to cells (75). The gradient allows for separation of cells in a mixed sample based on their density with monocytes collecting in a distinct middle band (76). Some groups use two rounds of cold aggregation; unfortunately, this method generates low number of monocytes (49). Cold aggregation followed by use of a Percoll^®^ gradient produces higher monocyte numbers than cold aggregation alone; however, monocyte viability can be significantly lower (50%) due to the prolonged isolation process and slight toxicity of the Percoll^®^ gradient reagents (49, 77). Many research groups have used the magnetic bead cell enrichment method in their immunological studies (49, 78, 79). There are two types of magnetic bead cell isolations: positive and negative selection (78). Positive selection isolates monocytes which present surface marker CD14 on their membrane using anti-CD14 magnetic beads. Although producing highly pure monocyte samples, binding to the beads may cause changes in cell function, activation and proliferation (80). Negative selection works by isolating immune populations other than monocytes. This method yields less pure cultures and is more prone to artifacts in immune responses (81). Despite the drawbacks related to magnetic bead isolation, monocyte enrichment using this method has the highest monocytic yields, viability, and cell purity when compared to cold aggregation and centrifugation on a Percoll^®^ density gradient (49).

Once monocytes have been isolated, they can be differentiated into DCs for use in IVI assays. Culture conditions for differentiation have been developed and optimized by several research groups (49, 78, 82, 83). In this approach, freshly isolated monocytes are cultured in the following media conditions to promote DC differentiation: RPMI media, L-glutamine, 10% serum, and exogenous cytokines, human interleukin 4 (IL-4) and granulocyte-macrophage colony-stimulating factor (GM-CSF). After 7 days of incubation in the differentiation condition, non-adherent cells, which are un-differentiated monocytes, can be removed (84). Culturing monocytes for more than eight days may generate less immunogenic MoDCs (50). There have been studies investigating the effects of concentration and type of cytokines, fetal bovine serum vs. horse serum, and cell culture plates vs. tubes on DC differentiation culture conditions. Overall, the combination of IL-4 and GM-CSF preserves the best conditions for generation of DCs with important phenotypic and functional characteristics such as high levels of CD1, major histocompatibility complex (MHC) class I and II, etc. (28, 78). There was no difference in DC morphology and differentiation when fetal bovine serum was replaced with horse serum, and MoDC culturing and differentiation shows slightly better yields when cultured in plates instead of tubes (49).

After differentiation via IL-4/GM-CSF stimulation in culture, MoDCs can be matured based on the needs of the researchers. Different research groups have tested various sets of cytokine and stimulatory molecule combinations to optimize efficient DC maturation. Addition of LPS, TNFα, IL-1ß, IL-6, and prostaglandin E_2_ (PGE_2_) can recreate the inflammatory state of a natural immune response, encouraging MoDC maturation (28, 41, 78, 85–89). Some research groups use cytokine media without PGE_2_, since PGE_2_ has an impact on DC production of IL-12 and DC migration (78, 90, 91). Maturation of MoDCs can be confirmed by LPS challenge and flow cytometry measurement of the DC maturation markers CD80, CD83, and CD86 (49, 74, 92). Once differentiated and matured, MoDCs do not proliferate extensively, with 98% of the cells staying in G0/G1 growth arrest (93, 94).

After differentiation and maturation, MoDCs are challenged with an antigen/adjuvant of interest to prepare for co-culture with T cells. This step serves to recapitulate the innate immune response in which DCs phagocytose and digest pathogenic antigens for presentation to the cells of the adaptive immune system (**Figure 1a**). Exposure to antigens typically lasts for an incubation period of 24 hours, after which cell supernatants can be collected and frozen at -80°C for later functional analysis for secreted molecules of the innate immune system. A portion of the MoDCs can also be collected and fixed for flow cytometry analysis (53).

#### 2.2.2 The dendritic cell-T cell interface assay

Once MoDCs have been challenged with the antigen or vaccine of interest, they are subsequently co-cultured with T cells to simulate DC induction of T cell activation and expansion (**Figures 1b, c**) (48, 95). Prior to DC:T cell co-culture, T cells can be isolated via positive selection using CD8^+^ and CD4^+^ microbeads from the same PBMC sample and frozen at –80 °C until use (**Figure 2b**) (53). Before freezing, T cells should be treated with an IL-2 and IL-7 cytokine cocktail to preserve T cell proliferation and differentiation of memory T cells, respectively (96, 97). Alternatively, the PBMCs can be cryopreserved and T cell isolation can be performed immediately prior to DC:T cell co-culture. T cells isolated from freshly isolated and cryopreserved PBMCs have been shown to yield similar results in DTI assays (96).

The success of DC:T cell co-culture is dependent on many factors. For instance, T cell expansion is proportional to DC numbers in culture (48), and the ratio of DCs to T cells is important in shaping the immune system response, with a 1:10 ratio being optimal to support T cell responses (98, 99). The optimal co-culture time is 48 hours, at which time activated T cells disengage from DCs and begin to proliferate (40). Choosing the right culturing plate is also important for successful DTI assay, with flat-bottom plates being preferred over round-bottom plates to decrease non-specific cellular interactions and to increase the magnitude of the T cell immune response (99, 100). After co-culture, supernatants can be collected for cytokine assays including enzyme-linked immunosorbent assays (ELISAs). Flow cytometry is commonly used to assess T cell activation, with CD3, CD4, and CD8 panels as general T cell markers (96). Specific markers of activation and proliferation are listed in the Readout section.

Due to the focused insight into communication between APCs of the innate immune system with T cells of the adaptive immune system, this assay set up has been widely applied in the field of immunology. The MoDC + DTI assay has been used to identify vaccine candidates and adjuvant combinations capable of enhanced activation and expansion of antigen-specific T cells (101) and can be applied to studying the differential responses from donors in various age groups (95, 102). Multiple research groups have used this system to characterize differences between newborn and adult immune responses (82, 83, 103). Other areas of research include cancer treatment (36, 92, 104), infectious diseases (105), and autoimmune diseases (106). The measurement of antigen-specific T cell responses could also advance therapeutic approaches to an individual patient - in the field of personalized medicine (96). Overall, MoDC assays are the industry standard for *in vitro* assays using DCs and are broadly used for clinical and experimental applications (107).

The MoDC + DTI assay is time-consuming due to the 10+ day cell culture period required (**Figure 2b**). This approach is also expensive due to the cost of procuring cytokines for differentiation that can suffer from batch-to-batch variability (78). As with all assays that rely on whole blood or PBMCs, donor-to-donor differences produce wide variation in DC yields from monocytes (49, 78). Low DC differentiation can be due to patient heterogeneity or the result of patient treatment or illness prior to the blood draw. Cytokine production can also vary between different donors (108), and individual donor responsiveness adds additional complexity to DTI optimization (99).

### 2.3 The microphysiological human tissue construct assay

The microphysiological human tissue construct (HTC) assay is the first human IVI assay that recapitulates some of the spatiotemporal interactions of the immune system. In this system, a confluent layer of human endothelial cells, such as human umbilical vein endothelial cells (HUVECs), is grown on top of a 3D extracellular matrix (ECM) substrate primarily made of collagen (**Figure 2c**) for 7–10 days prior to start of the HTC assay (109). The use of single-cell epithelium is critical to mimic monocyte migration through a human capillary vein in response to a pathogen or vaccine stimulus (110–113). The established confluent endothelial layer in conjunction with the collagen-based ECM is referred to as a human tissue construct (TC). Monocytes purified from PBMCs are added to the top of the TC and allowed to autonomously transmigrate into the TC for 1.5 hr, after which the non-extravasated monocytes from the top layer are removed. At this point, the extravasated monocytes are exposed to a vaccine or antigen of choice and cultured for 48 hr. During this time, monocytes autonomously differentiate into MoDCs as they reverse transmigrate through the endothelial layer in response to the antigen. This process mimics migration of APCs from tissues into the lymphatic system (114, 115). About half of the monocytes remain in the ECM and do not differentiate into DCs (112). The mixture of immature and mature DCs from the top of the culture system can be removed to co-culture with autologous T cells isolated from the same PBMC sample. T cell proliferation confirms successful stimulation in co-culture, and media supernatant can be collected and analyzed for cytokine/chemokine production.

The HTC model enables monocytic self-differentiation into migratory DCs in response to a challenge. This model does not require the addition of exogenous cytokines (109). The HTC assay integrates both innate and adaptive immune responses while maintaining the sequential timing of key cellular interactions. The use of PBMCs from the same donor for each step supports the identification of donor-specific immune responses (116). This model mimics the physiology of the immune system more closely and avoids DC differentiation to artificial phenotypes that are not seen in humans (41, 117). Sanchez-Schmitz et al. were the first to develop the HTC assay and measure a CD4^+^ T cell response to immunization that matches the results seen in patients (109). The HTC model is a promising system to accelerate vaccine development since the same patient sample can be tested both with and without vaccination, responses can be simultaneously examined from a variety of populations (e.g., neonates, elderly, immunocompromised), and costs are significantly reduced compared to clinical trials (109, 118).

The Modular Immune *in vitro* Construct (MIMIC^®^) system is an advanced HTC-based assay that seeks to recapitulate the innate and adaptive immune responses as true to the *in vivo* process as possible. Like the HTC, this system involves a collagen membrane and HUVEC endothelial layer; however, the TC is established in the insert of a transwell plate instead of within the wells of a standard cell culture plate. MIMIC^®^ consists of three modules: 1) the Peripheral Tissue Equivalent (PTE) module; 2) the Lymphoid Tissue Equivalent (LTE) module; and 3) the Functional Assay construct (119). The PTE module mimics innate system responses and allows for testing of vaccines, antigens, adjuvants, chemicals, and therapeutical compounds. In the PTE, PBMCs are added to the TC established in the transwell insert and allowed to transmigrate and differentiate into DCs as in the HTC. DCs can be challenged at any point during their trans or reverse transmigration (119). The LTE component mimics the adaptive immune system in the lymph nodes where DCs interact with T and B cells (108, 118, 119). Immune cells are added at specific time points to reflect human cell interactions within lymph nodes. DCs are co-cultured with T cells to recapitulate physiological conditions within lymph nodes. B cells are added to the DC:T cell co-culture 1–3 days later to increase B cell antibody response (119). Lastly, in the Functional Assay module, the immune response is assessed by traditional methods such as ELISAs and new techniques designed to measure the magnitude, specificity, and affinity of antibodies generated in a MIMIC^®^ experiment (119). Presence of dendritic cells, monocytes, and lymphocytes in the MIMIC system was confirmed by flow cytometry (108). MIMIC^®^ is indeed modular; one or more of the modules can be performed alone or in combination with the others (120). Many of these steps have been automated using a robotic arm to make it less labor-intensive and increase throughput (4-times faster than a human researcher) (119). For a comprehensive review of the MIMIC^®^ system, see the review written by Drake et al. in 2012.

The limitations of HTCs as an IVI assay are similar to those of the MoDC + DTI assay, including the complexity of assays requiring extensive expertise to perform, and time it takes to complete the assay. The incorporation of a robotic arm in the MIMIC system alleviates some of the hands-on time required, however it is an expensive addition. While the HTC seeks to mimic the spatiotemporal features of the human immune response, it does not successfully mimic physiologic blood flow or all the migratory cell transit events that happen during the immune response.

## 3 Readouts

### 3.1 Cell surface marker expression

**Figure 3:**
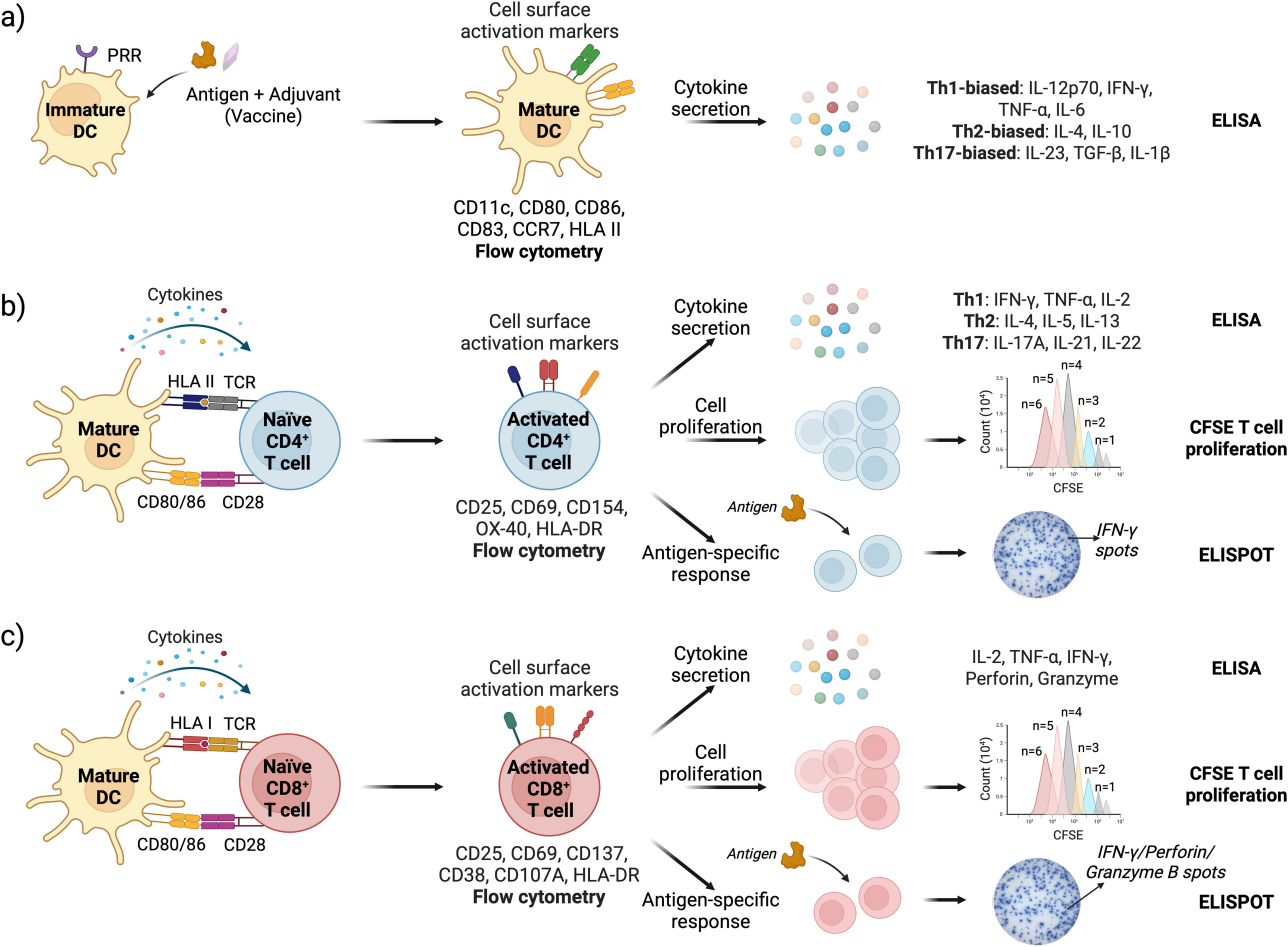
Schematic representation of DC and T cell activation assays with readouts; **(a)** Vaccine stimulation activates DCs leading to upregulation of cell surface markers (assessed by flow cytometry) and cytokine secretion (measured by ELISA); **(b)** DC-CD4^+^ T cell and **(c)** DC-CD8^+^ T cell interface assays are used to assess T cell activation, proliferation, and antigen-specific immune responses. Cell surface activation markers and cytokine response are analyzed by flow cytometry and ELISA, respectively. T cell proliferation is quantified by tracking CFSE dye dilution in cells using flow cytometry. Antigen-specific responses are measured via ELISPOT, specifically detecting IFN-γ (for both CD4^+^ and CD8^+^ T cells), and Granzyme B and Perforin specifically for CD8 T cells (162–165).

Flow cytometry is the most commonly used method to evaluate the activation status of DCs and T cells in IVI assays. For DCs, the upregulation of costimulatory molecules CD80 and CD86, along with the maturation marker CD83, chemokine receptor CCR7, and human leukocyte antigen (HLA) class II antigens, indicates their transition from an immature to a mature phenotype (78) (**Figure 3a**). Activation of CD4^+^ helper T cells and CD8^+^ cytotoxic T cells can be determined by monitoring changes in the surface expression of CD25 (IL-2Rα), CD69, CD71, OX40, and HLA-DR. Additionally, CD154 (CD40L) serves as a key activation marker for CD4^+^ T cells, while CD137 (4-1BB) is a marker of activated CD8^+^ cytotoxic T cells (121) (**Figures 3b, c**).

### 3.2 Cytokine profile

Upon activation, DCs and T cells produce and secrete cytokines, which serve as key signaling and effector molecules. Several methods are available to measure cytokine secretion, with ELISA being the most widely used due to its ease of use and moderate throughput, particularly when quantifying a limited number of cytokines. Multiplexed bead-based assays such as Luminex offer significantly higher throughput, allowing simultaneous quantification of multiple cytokines (122). It is preferred for a more comprehensive analysis of cytokine responses induced by an antigen or vaccine construct. Intracellular cytokine staining (ICS) followed by flow cytometry can be used to assess cytokine production at the single-cell level (123). Additionally, the enzyme-linked immunospot (ELISPOT) assay enables the detection of cytokine-producing T cells in response to specific antigens (124).

The DC cytokine secretome provides insight into T cell polarization induced by an antigen or a vaccine (125–127). High levels of IL-12p70 and IFN-γ, along with pro-inflammatory cytokines TNF-α and IL-6, indicate Th1-biased polarization, which supports cell-mediated immunity and antiviral responses. Elevated IL-4, along with IL-10, promotes Th2 polarization, favoring humoral immunity. Th17-biased polarization is driven by IL-23, TGF-β, and IL-1β, which support mucosal immunity and neutrophil recruitment (**Figure 3a**). Like DCs, successful CD4^+^ T cell activation results in high levels of IFN-γ, IL-2, and TNF-α for Th1 cells; IL-4, IL-5, and IL-13 for Th2 cells; and IL-17A, IL-21, and IL-22 for Th17 cells (128) (**Figure 3b**). Activated cytotoxic CD8^+^ T cells produce high levels of Granzyme B and Perforin as part of their target cell-killing mechanism (**Figure 3c**).

### 3.3 Gene expression profile

Flow cytometry and ELISA, which measure functional protein levels, can be complemented with reverse transcription quantitative PCR (RT-qPCR) to determine changes in gene expression profile in response to an antigen or a vaccine (125, 129). With high sensitivity and specificity, RT-qPCR allows high throughput analysis of low-abundance transcripts and early-stage immune responses. However, RT-qPCR requires additional sample processing to extract and purify cellular RNA following immune cell activation procedure, making it more time- and labor-intensive compared to protein quantification assays.

### 3.4 Dendritic cell endocytosis assays

As part of their innate immune sentinel function, DCs continuously survey their environment and capture pathogens and apoptotic cells through endocytosis. The endocytic and phagocytic activities of immature DCs are crucial to elicit a strong innate immune response, which subsequently drives a robust antigen-specific adaptive response. Several methods can be employed to measure cellular uptake in immature DCs; among these, FITC-dextran uptake assay and bead-based phagocytosis assay are frequently used (130–132). A critical consideration in these assays is that DCs naturally downregulate their endocytic activity as they mature. To ensure accurate results, care must be taken to avoid endotoxin contamination in these assays, as endotoxins can trigger premature DC maturation, leading to reduced endocytic activity and potential misinterpretation of the results.

### 3.5 T cell proliferation assays

Upon encountering a foreign antigen presented by activated DCs, CD4^+^ and CD8^+^ T cells get activated and undergo rapid proliferation. The most widely used method to determine T cell proliferation involves labeling T cells with cell proliferation dyes such as carboxyfluorescein succinimidyl ester (CFSE), CellTrace Violet, and eFluor 670, and then co-culturing them with activated DCs. As the T cells divide, the dye molecules incorporated in the cells get partitioned between the daughter cells, leading to their progressive ‘dilution’ in the cells. Flow cytometry is then used to analyze the fluorescence intensity – progressive decrease in the fluorescence intensity indicates each subsequent cell division (133) (**Figures 3b, c**). Other T cell proliferation assays include ^3^H-thymidine incorporation and BrdU or EdU incorporation, which measures DNA synthesis and incorporation of either radioactive ^3^H-thymidine or thymidine analogues BrdU or EdU that can be detected using BrdU- or EdU-specific antibodies (134). Ki-67 staining identifies T cell proliferation by measuring Ki-67 protein, which is expressed during cell division (135).

### 3.6 T cell antigen sp*ecificity assays*

The antigen specificity of activated T cells is a critical determinant of the quality of immune protection elicited by a vaccine. In IVI assays, the antigen specificity of T cells can be determined either directly after co-culture with antigen-pulsed DCs or following a subsequent restimulation with antigen-pulsed DCs. For direct identification without restimulation, peptide-HLA tetramer staining offers high sensitivity and selectivity (136, 137). However, the method is very costly as tetramer needs to be custom-made for the donor’s HLA alleles and specific antigenic peptide sequences derived from the whole protein antigen. Restimulation assays typically lead to more robust T cell activity and ensure that the T cell activation was indeed antigen-specific (138). Post restimulation with antigen-pulsed DCs or peptides, T cell functions can be measured using proliferation assay, intracellular cytokine staining, ELISPOT assay to primarily measure IFN-γ production, and measuring expression of cell surface markers such as CD154 for CD4 T helper cells and CD137 and CD107A (degranulation marker) for CD8^+^ cytotoxic T cells using flow cytometry (**Figures 3b, c**).

The three types of IVI assays described above vary in complexity, and with increasing complexity, they also provide progressively more specific insights into immune function and the cellular responses to vaccines. It is important to note that not all the readouts described in this Section are equally applicable or biologically informative across all IVI assays. For example, analysis of cytokine profile in a whole blood or PBMC assay can provide a broad immunogenicity profile of the vaccines. However, more granular analyses such as DC or T cell activation are limited for these assays due to low abundance of DCs in peripheral blood and the absence of spatiotemporal dynamics critical for T cell activation. Similarly, assays for evaluating DC endocytosis and T cell proliferation are not well-suited to whole blood or PBMC assay. Moreover, there are differences in the strength of the immune response between different IVI assays for various adjuvants and immunomodulators (118). More complex IVI assays provide higher immune responses (108, 139). Researchers who developed the MIMIC platform claimed that its complexity contributes significantly to receiving more physiologically-accurate immune responses that recapitulate complexity of immune responses <i>in vivo and in the clinic (118, 120). Therefore, we emphasize that the choice of assay readouts must be made in conjunction with the selection of the IVI assay based on specific research question being addressed.

## 4 Comparing *in vivo* and *in vitro* models for vaccine development

IVI assays and *in vivo* animal pre-clinical studies serve as complementary tools in the vaccine development pipeline. Pre-clinical animal models such as mice and non-human primates (NHPs) remain the gold standard for evaluating vaccine safety, immunogenicity, and efficacy, providing a whole-organism context that encompasses immune, metabolic, neurological, and toxicological responses. However, it must be recognized that physiological and immunological differences between animal models and humans can limit the direct translatability of pre-clinical findings. For instance, mice have a significantly higher proportion of lymphocytes (70–90%) in peripheral blood compared to humans (30–50%) (140). Beyond this, fundamental differences in the functionality of both innate and adaptive immune systems can reduce the predictive value of mouse models. Even NHPs, despite their phylogenetic proximity to humans, exhibit key immunological distinctions that may lead to differential vaccine responses (141, 142). This is evident in cases where vaccine candidates that performed well in animal studies failed to demonstrate efficacy in human trials. Notable examples include the Dengvaxia vaccine (143), which showed promising results in NHPs but exacerbated disease severity in seronegative individuals during Phase 3 human trials, and an adenovirus-based HIV-1 vaccine, which failed in Phase 2b trials due to unidentified HLA allele-epitope mismatch between NHPs and humans (144). While *in vivo* studies are indispensable, IVI assays offer critical insights that animal models cannot provide. IVI assays enable mechanistic assessment of antigen presentation, cellular activation, and cytokine profiles. They are particularly valuable for studying the impact of HLA diversity, age, sex, chronic conditions (e.g., diabetes), and prior antigen exposure on vaccine response. For example, IVI assays have been used to identify effective adjuvant combinations for neonates, adults, and elderly populations (101), and to study age-specific immune responses to COVID-19 mRNA vaccines (145) and influenza vaccine (120). Importantly, IVI assays are not intended to replace *in vivo* studies but to complement them. By validating *in vivo* findings, IVI assays can help bridge the gap between animal data and human clinical outcomes. They also provide valuable mechanistic data that can support the identification of correlates or surrogates of protection, which are critical for evaluating vaccine efficacy in clinical trials. We propose that an integrated approach, combining the strengths of *in vivo* animal models and human IVI assays as elaborated below, offers a more robust and predictive framework for vaccine development. Such an approach enables early, informed decision-making checkpoints, reducing reliance on animal models where possible, enhancing translational relevance, and ultimately accelerating the path to safe and effective vaccines.

*In vivo* models allow researchers to observe the complex network of biological interactions in their natural environment, provide physiological relevance, and allow for long-term observation (6, 146, 147). The most common *in vivo* animal model in immunology studies is the mouse model (6); however, due to the inherent differences between mice and humans, there have been significant advances in the development of a humanized mouse model to better recapitulate the human immune system (148, 149). Humanized mouse models are immunodeficient mice that have received human cell or tissue transplants, including PBMCs, hematopoietic stem cells, bone marrow, liver cells, and thymus cells (150, 151). Humanized mouse research contributes significantly to studying human infectious diseases, immunity, cancer, therapies, and medicine (152, 153), but there are still improvements to be made to this animal model, including improved recapitulation of the human B cell response (151). The major drawbacks of *in vivo* animal models are individual variability, high cost, availability of specialized animals, and inability to accurately recapitulate human responses (154). Vaccine candidates that provided sufficient immune responses in animal models can sometimes demonstrate reduced efficacy and safety in subsequent clinical studies, underscoring the need to bridge the gap between pre-clinical *in vivo* studies and human immune responses (62).There is also a trend in the scientific community to replace, reduce, and refine animal testing due to ethical concerns (154–156).

*In vitro* platforms allow researchers to characterize the interactions of a pathogen or an antigen with human immune cells at the cellular and molecular levels under controlled conditions. The controlled environment permits rigorous hypothesis testing, including testing of different vaccine components (adjuvants, antigens, etc.) to distinguish their interplay and individual effects. These models are cost- and time-efficient, scalable, and can account for previous immunological exposures (96, 119, 120). It is standard practice for vaccine candidates to be tested in two animal systems before moving on to human studies (120). *In vitro* platforms can speed up this process and offer higher throughput (157). *In vitro* experiments also use fewer resources and are less ethically concerning. The limitations of human *in vitro* platforms when compared to animal models include the inability to compare different vaccination administration routes (i.e. oral, subcutaneous, intranasal, intramuscular) on the immune response (108, 120, 158). *In vitro* platforms cannot account for other complex interactions within the human body, such as effects of the gut microbiome on the immune cellular interactions (159). While it is difficult for human *in vitro* platforms to capture the complex interactions between different organ systems, they can accelerate the process of vaccine development and save costs, time, and animal sacrifice for optimization purposes. They provide information about vaccine potential using high-throughput screening. IVI assays can be crucial for rapid vaccine approval and release in desperate times including pandemics (160). Human *in vitro* platforms can precede animal studies to reduce the number of experimental animals and testing of different treatments (161). This approach allows selection of vaccine candidates for pre-clinical evaluation in a quick, reliable, cost-effective, and safe manner (160). IVI assays are an important pre-clinical tool to assess the safety of vaccine formulations. By using *in vitro* models in conjunction with *in vivo* models, more definitive results can be generated, since biological processes within intact organisms (*in vivo* models) and molecular/cellular mechanisms (*in vitro* platforms) can be captured.

## 5 Conclusions and future directions

Human IVI assays provide a rapid and cost-effective assessment of both innate and adaptive immune responses to emerging pathogens. These platforms facilitate the identification of promising vaccine candidates, eliminating those that cause unintended toxicity, and reducing reliance on animal models. IVI assays also enable evaluation of immune responses across diverse populations considering factors such as age, HLA type, prior exposure, and disease state (108, 120). Their impact extends to both basic and translational research, driving innovation in immunology and vaccine development (41).

Each *in vitro* platform has distinct advantages and challenges (**Table 1**). Advances in immunological and biomedical research continue to enhance IVI assays, improving their ability to replicate the complexity of human immune responses (41). Currently, a combination of *in vitro* and *in vivo* studies is required for pre-clinical testing and vaccine development. However, with the emergence of novel high-throughput platforms like MIMIC, IVI assays are increasingly capable of replicating clinical vaccine reactogenicity (108). IVI assays have the potential to revolutionize vaccine development, streamlining the down-selection of vaccine candidates, reducing the need for animal testing, and accelerating the pathway to clinical trials.
